# Immunoglobulin G4-Related Disease Presenting as Recurrent Acute Pancreatitis

**DOI:** 10.7759/cureus.68844

**Published:** 2024-09-07

**Authors:** Vaibhavi A Gor, Krutik J Brahmbhatt, Nachiket Patel, Neel R Vaidya, Vasuprada Narasimhan, Divya A Mehta

**Affiliations:** 1 Medicine, Government Medical College, Vadodara, IND; 2 Medicine, GMERS (Gujarat Medical Education and Research Society) Medical College, Gandhinagar, IND

**Keywords:** autoimmune pancreatitis, complicated acute pancreatitis, igg4 -related disease, pseudo cyst, recurrent acute pancreatitis, rheumatology & autoimmune diseases

## Abstract

Immunoglobulin G4-related disease (IgG4-RD) is a rare immune-mediated disease affecting multiple organs and tissues. There is often the presence of elevated serum Ig4 subtype with histological evidence of lymphoplasmacytic infiltration, fibrosis, and phlebitis. The mainstay of treatment is steroid therapy.

This case report is based on a 24-year-old man with IgG4-related type 1 autoimmune pancreatitis (AIP) who also had elevated serum IgG4 subclass and histological features in keeping with IgG4-RD. The main complaints were dry cough, nasal congestion with sneezing, sore throat, and fever. Necessary investigations were performed and based on the International Consensus Diagnostic Criteria, the diagnosis of AIP type 1 was confirmed, which is a pancreatic manifestation of IgG4-RD. He was subsequently treated with prednisolone and azathioprine and is showing a good response to the treatment.

## Introduction

Immunoglobulin G4-related disease (IgG4-RD) is a rare and recently recognized immune-mediated disorder. A set of rare fibro-inflammatory illnesses affecting many organ systems having similar pathologic characteristics, such as the noticeable predominance of plasma cells that secrete the immunoglobulin subtype IgG4. Most patients also have elevated serum IgG4.

These organ systems are many and include the kidney (tubulointerstitial nephritis), lungs (parenchyma or pleura), pancreas, orbit (including the lacrimal gland, extraocular muscles, and/or other soft tissues producing an orbital pseudotumor), salivary glands, meninges, pituitary, thyroid, lymph nodes, gall bladder, liver, breast, prostate, skin, peripheral nerve, pericardium, mediastinum, retroperitoneum, aorta, and large arteries. The commonest clinical manifestations are lymphadenopathy, submandibular gland enlargement, and autoimmune pancreatitis (AIP) [1], but since IgG4-RD is a systemic disease, it can affect almost any organ. In 2014, the Japanese team published comprehensive diagnostic criteria for IgG4-RD. The diagnostic criteria are based on: (i) organ enlargement, mass or nodular lesions, or organ dysfunction; (ii) increased serum IgG4 concentration >135 ​mg/dl, and (iii) infiltration of IgG4+ cells (>10 ​cells/high power field (HPF) and IgG4+/IgG+ cell ratio >40%). Patients who fulfill the organ-specific criteria (i,ii,iii) have a definite diagnosis. A diagnosis of IgG4-RD is possible in patients who fulfill criteria (i) and (ii), but with negative results on histopathology or without histopathologic examination, whereas a diagnosis of IgG4-RD is probable in patients with organ involvement (i), but without increased IgG4 concentration. We are reporting a 24-year-old man with IgG4-related type 1 autoimmune pancreatitis (AIP). He also had elevated serum IgG4 subclass and histological features consistent with IgG4-RD [2].

## Case presentation

**Figure 1:**
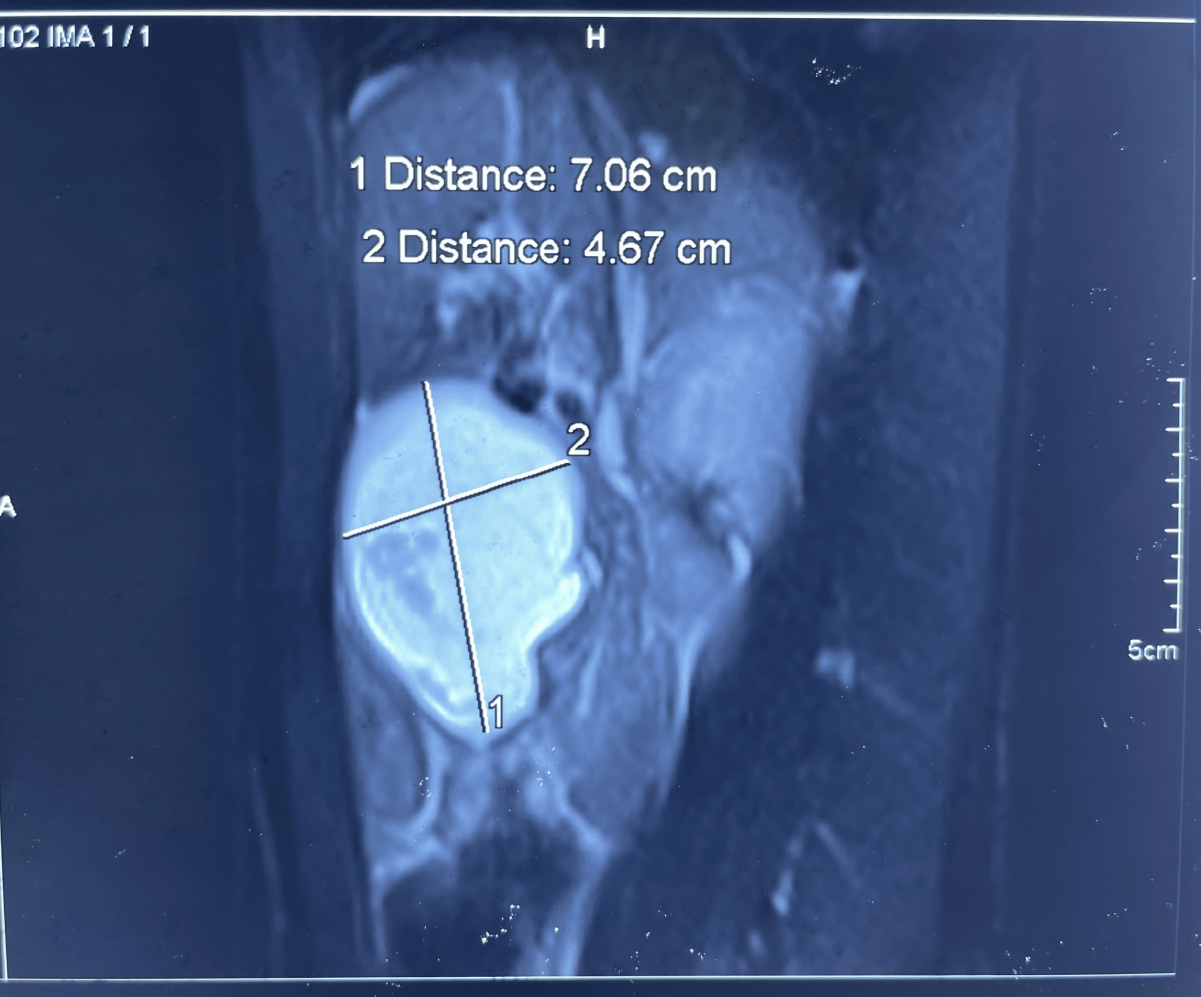
Multidetector computed tomography (MDCT) scan during the second episode of acute pancreatitis

A 24-year-old male presented with chief complaints of dry cough, nasal congestion with sneezing, sore throat, and fever for 10 days. The fever was low-grade with chills and was relieved on medication. There was no complaint of pain, vomiting, diarrhea, or breathlessness. We treated him for acute pharyngitis. On eliciting past history, the patient had nine episodes of acute pancreatitis in the past three years. Amylase and lipase during each episode were elevated beyond 1000 U/L. The patient was managed conservatively and was discharged after 10 days of ICU stay. During the second episode (which happened three months after the first episode), the severity of pain increased, and multidetector computed tomography (MDCT) showed pseudocyst in relation to the proximal part of the body of the pancreas (Figure 1).

**Figure 2:**
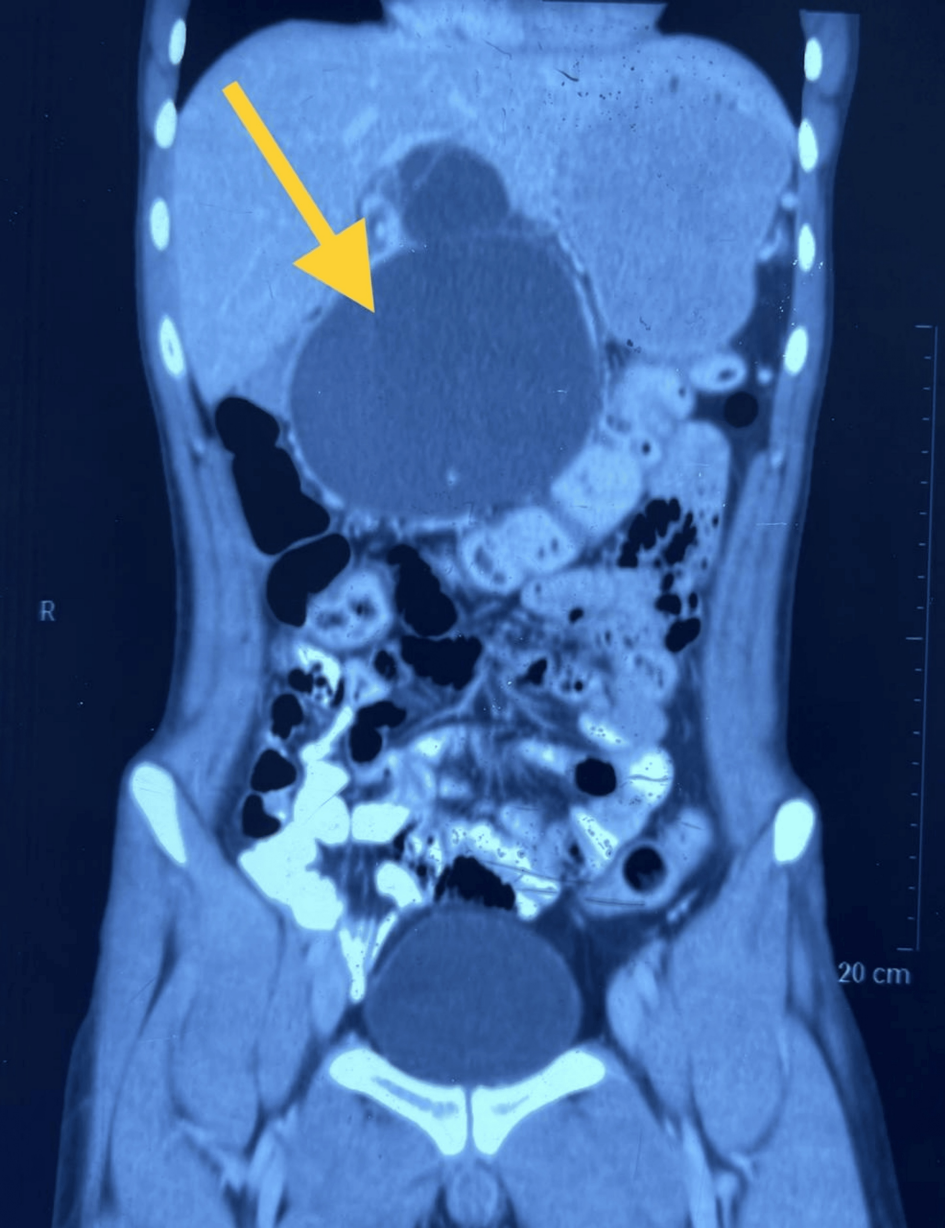
Multidetector computed tomography (MDCT) scan of the abdomen before cystogastrostomy in coronal section showing pancreatic pseudocyst measuring 123*99*73 mm (yellow arrow)

**Figure 3:**
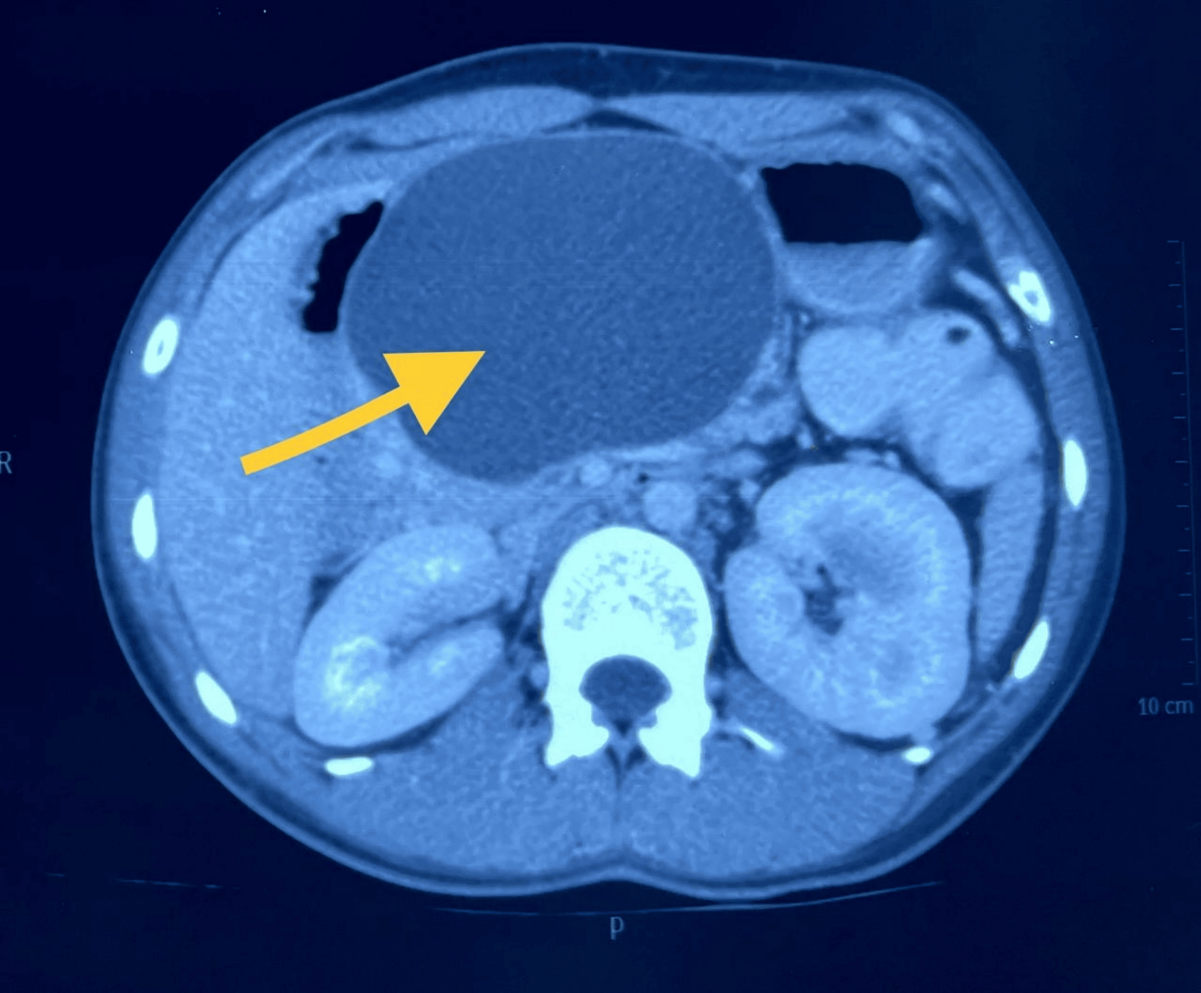
Pancreatic pseudocyst (yellow arrow) of the abdomen in axial view in computed tomography (CT) scan

The patient was again managed conservatively and was discharged. He remained asymptomatic for the next five months and then was again admitted for acute pancreatitis wherein the size of the pseudo cyst increased (Figures 2, 3). He was operated by cystogastrostomy [3].

Since then, the patient had six more episodes of acute pancreatitis every three to four months and was managed conservatively in a local hospital. Since the last 1.5 years, he complained of increasing fatigue in day-to-day activities and has lost 7 kg in the past one year. He also complains of repeated attacks of dry cough and cold, especially in winters, and occasional dull aching abdominal pain, especially after heavy meals. No similar family history was present. There is no history of dyslipidemia and diabetes in his parents. The patient denies any substance abuse.

On physical examination, temperature was raised to 100.4°F, pulse rate was 106/min, respiratory rate was 18/min, and blood pressure was 110/80 mmHg. Congestion was noted in post-pharyngeal wall with occasional scattered expiratory rhonchi on auscultation. Cardiovascular, central nervous system (CNS), and abdomen examinations were normal. The results of the routine hematological investigation is shown in Table 1 and blood chemistry investigation results are show in Table 2. The patient was treated for acute pharyngitis. 

**Table 1:** Hematological report of the patient RBC: Red blood cell; PCV: packed cell volume; MCV: mean corpuscular volume; MCHC: mean corpuscular hemoglobin concentration; WBC: white blood cell; ESR: erythrocyte sedimentation rate

PARAMETERS	RESULTS	REFERENCE RANGE
Hemoglobin	11.20 g/dL	12-16 g/dL
RBC	4.85* 10^6	4.50-5.50* 10^6
PCV	36.90%	40-50%
MCV	76.10 fL	80-98 fL
MCH	23.10 pg	28-32 pg
MCHC	30.40 g/dL	33-36 g/dL
Platelets	4.16* 10^6/ L	1.50-4.50* 10^6/L
WBC	14800/mm^3^	4500-1000
Neutrophils	76%	50-70%
Lymphocytes	21%	20-40%
Monocytes	2%	2-8%
Eosinophils	1%	0-6%
ESR	132 mm/h	0-10 mm/h

**Table 2:** Blood chemistry results of the patient AST: Aspartate aminotransferase; ALT: alanine aminotransferase; HDL: high-density lipoprotein; LDL: low-density lipoprotein; VLDL: very low-density lipoprotein; TSH: thyroid stimulating hormone

INVESTIGATION	RESULTS	REFERENCE RANGE
Total bilirubin	0.7 mg/dL	0.3-1.0 mg/dL
Direct bilirubin	0.3 mg/dL	0.1-0.3 mg/dL
Indirect bilirubin	0.4 mg/dL	0.2-0.8 mg/dL
AST	25 U/L	12-38 U/L
ALT	25 U/L	10-40 U/L
Serum amylase	40 U/L	30-110 U/L
Serum lipase	41.3 U/L	0-160 U/L
Serum urea	23 mg/dL	6-24 mg/dL
Serum creatinine	0.95 mg/dL	0.7-1.3 mg/dL
Serum cholesterol	155 mg/dL	<200 mg/dL
Serum HDL	36 mg/dL	40-60 mg/dL
Serum LDL	103 mg/dL	<100 mg/dL
Serum VLDL	15 mg/dL	2-30 mg/dL
Serum TSH	2.14 mU/L	0.5-4.0 mU/L
Serum sodium	135 mEq/L	136-145 mEq/L
Serum potassium	4.20 mEq/L	3.5-5.0 mEq/L
Serum calcium	9.5 mEq/L	8.5-10.2 mEq/L

As the patient had repeated episodes of acute pancreatitis, tests to diagnose the cause of recurrent pancreatitis were done as shown in Table 1. His serum calcium and lipid profile were normal. IGg4 levels were >3.93g/L. Histopathology report of the tissue removed during cystogastrostomy was collected from the previous hospital, which showed mixed inflammatory infiltration (lymphocytes with plasma cells) with fibro-collagenous deposition. IGg4 staining of the tissue was not done at that time. The patient was treated with steroids: prednisolone 1mg/kg (40 mg) for four weeks, then tapering over the next four weeks with 10 mg initially and eventually adding azathioprine 50 mg OD in maintenance when the patient developed an episode of pancreatitis on steroid only. Since then, he has progressively improved with normal appetite and has had no episode of acute pancreatitis, and his erythrocyte sedimentation rate (ESR) dropped from 132 mm/h to 18 mm/h.

## Discussion

Autoimmune pancreatitis (AIP) is a rare cause of chronic pancreatitis [4]. Presently two types are identified: type 1 and type 2 (idiopathic duct centric chronic pancreatitis). Type 1 AIP is associated with IgG4-R, more common in males, with 66% of the cases associated with serum IgG4 elevation and 50% of the patients showing other organ involvement [5]. Histology shows lymphoplasmacytic infiltration, periodical inflammation, storiform fibrosis and obliterative phlebitis [6]. IgG4 staining of the tissue shows more than 10 cells/hpf. Type 2 AIP is not associated with IgG4-RD, has equal incidence in males and females, with less organ involvement and histology showing granulocytic epithelial lesions (GEL). Various diagnostic criteria are developed in International Consensus Diagnostic Criteria, which include serological testing (elevated serum IgG4 levels), histological findings, radiological imaging findings like focal or diffuse enlargement due to active disease or gland atrophied due to previous disease, presence of inflammatory rim sign and capsule sign on computed tomography (CT). Both types respond to steroids. Glucocorticoids [7] have shown efficacy in alleviating symptoms, decreasing the size of the pancreas and reversing the histopathology features in patients with AIP. Patients typically respond dramatically to glucocorticoid therapy within a two- to four-week period. Clinical remission was achieved in 99% of type 1 AIP and 92% of type 2 AIP patients with steroids. However, disease relapse occurred in 31% and 9% of patients with type 1 and type 2 AIP, respectively. Patients with multiple relapses may be managed with an immunomodulator (e.g. azathioprine, 6-mercaptopurine, or mycophenolate mofetil) or B-cell depletion therapy (e.g. rituximab) [8]. Significant adverse effects from azathioprine include pancreatitis, gastrointestinal problems (vomiting and nausea), liver damage, severe leukopenia, and hair loss. Although the prevalence of azathioprine-induced pancreatitis in AIP patients is unknown, the risk of acute pancreatitis after azathioprine medication is comparatively low. In clinical practice, it might be challenging to diagnose AIP relapses or adverse effects from azathioprine when a patient with AIP develops pancreatitis while receiving treatment. A recent analysis suggests that the HLA-DQA1-HLA-DRB1 polymorphism is a significant marker for the risk of azathioprine-induced pancreatitis. Thus, an analysis of human leukocyte antigens (HLA) type would be beneficial to prevent azathioprine-induced pancreatitis in Caucasians [9].

## Conclusions

In conclusion, IGg4-related disease is an immune-mediated fibro-inflammatory condition, having a wide range of clinical manifestations. Our patient had multiple episodes of acute pancreatitis, so he was evaluated for that condition. We ruled out hypertriglyceridemia and hypercalcemia, keeping a differential of autoimmune pancreatitis type 1, which is a pancreatic manifestation of IgG4-RD. We looked for serum IgG4 levels, which were elevated. It was associated with atrophic pancreas on imaging and histopathology showed lympho-plasmacytic infiltration with a fibro-collagenous deposition. The patient was treated with steroids: prednisolone 1mg/kg (40 mg) for four weeks, then tapering over the next four weeks with 10 mg initially, and azathioprine 50 mg OD added subsequently as a maintenance dose. Since then, he has progressively improved and has had no episodes of acute pancreatitis since then. His ESR improved from 132 mm/h to 18 mm/h.
